# Social Determinants, Mental Well-Being, and Disrupted Life Transitions Among Young Adults with Disabling Mental Health Conditions

**DOI:** 10.1007/s11414-024-09924-0

**Published:** 2025-01-13

**Authors:** Judith A. Cook, Jessica A. Jonikas, Jane K. Burke-Miller, Frances Aranda, Michelle G. Mullen, Maryann Davis, Kathryn Sabella

**Affiliations:** 1 Center on Mental Health Services Research and Policy, Department of Psychiatry, University of Illinois Chicago College of Medicine, 1601 West Taylor Street, 4th Floor, M/C 912, Chicago, IL 60612 USA; 2 Transitions to Adulthood Center for Research, Department of Psychiatry, UMass Chan Medical Schoo, 222 Maple Avenue, Shrewsbury, MA 01545 USA

## Abstract

This study sought to understand how young adults (age 18–25) with histories of mental health disorders are coping with disrupted transitions to adulthood during the COVID-19 pandemic. A cross-sectional web survey was conducted in March–June 2021 of 967 US young adults with pre-pandemic psychiatric disability to assess their current psychiatric status, interrupted transitions, and associations with social determinants including income, community participation, and social context. Mental health was assessed with the Patient Health Questionnaire (PHQ-9), Generalized Anxiety Disorder Scale (GAD-7), and PTSD Checklist-Civilian Version. Social determinants were identified with the Epidemic-Pandemic Impacts Inventory. Interrupted transitions were measured with the Young Adult Disrupted Transitions Assessment. Multivariable logistic regression models predicted four types of transition disruptions and associations with current mental health, social determinants, and demographic factors. Disruptions were reported by 81.1% including interrupted education completion (38.3%), employment careers (37.6%), residential independence (27.7%), and intimate partner relationships (22.9%). Many screened positive for major depressive disorder (81.7%), PTSD (85.5%), or GAD (58.6%). Disruption in establishing intimate partner relationships was associated with depression, anxiety, and PTSD. Interrupted residential independence was associated with anxiety. Interrupted education completion was associated with PTSD. Interrupted employment was associated with anxiety. Social determinants significant in these models included social connections, community participation, income, and racial/ethnic identification. Results illuminate ways that current mental health and social determinants affect transition interruptions during the pandemic. Findings suggest the need for interdisciplinary approaches, integrated models of care, and assistance accessing treatment, rehabilitation, and community support services from adult service systems.

**Supplementary Information:**

The online version contains supplementary material available at 10.1007/s11414-024-09924-0.

## Introduction

Research over the past few decades has called attention to the new life stage of emerging adulthood and its implications for mental health.^1^ Stress caused by demands of the transition to adulthood can lead to the onset of mental health disorders or exacerbation of pre-existing disorders.^2,3^ Almost a tenth (9.7%) of young people in their transition years (age 18 to 25) experience serious mental illness,^4^ defined as a mental health condition accompanied by moderate to severe impairment in daily living.^5^ This can cause delays in young people’s cognitive, social, and emotional development that interfere with transitions such as completing formal education, establishing residential independence, and building a career.^6^ These transitions are complicated not only by the need to manage serious mental health conditions, but also by the need to navigate unfamiliar adult service delivery systems^7,8^ within the context of social determinants including economic security, social context, and community participation.^9^

Studies show that young people’s mental health is both a cause and consequence of disrupted transitions,^10,11^ yet no research could be located that focused on the impact of the COVID-19 pandemic on young people with disabling mental health conditions.^12,13^ While a considerable literature documents the pandemic’s negative effects on the mental health of emerging adults in general^14–16^ only a handful of studies address pandemic impacts specifically on transition experiences, and none of these focus on young adults with pre-pandemic mental health conditions. This is so despite concerns that people with pre-existing mental health disorders are especially at-risk for poor pandemic outcomes.^17,18^

Research findings regarding pandemic experiences of young adults in the general population are instructive. In a cohort of Irish young adults in their early twenties surveyed pre- and post-pandemic,^19^ a large majority experienced disruption to their employment, education, and social activities. Notably, educational disruption was associated with inability to engage in sports and social activities in this cohort, and employment disruption was associated with poor diet and junk food consumption.^19^ Studies of young people in the USA reporting discomfort arising from living with parents during the pandemic^20,21^ found worsening mental health linked to loss of autonomy and poorer parent–child interactions. An online survey of psychological adjustment to pandemic-related life challenges reported by 180 US young adults found an association between job-related problems and sleep disruption.^22^ Finally, a qualitative study of close relationships during the pandemic reported by 707 US college students found that negative changes in romantic relationships were associated with both physical and emotional distancing due to isolation and loneliness.^23^

Given the foregoing, the purpose of this study was to identify the prevalence of disrupted transitions in a group of community-dwelling young adults with disabling mental health conditions. The aim was to explore associations between disrupted transitions and poor current mental health, defined as screening positive for major depressive disorder (MDD), generalized anxiety disorder (GAD), and post-traumatic stress disorder (PTSD). The researchers hypothesized that linkages between poor mental health and transition disruptions would persist, even controlling for covariates known to influence transition experiences more generally, as well as social determinants during the pandemic.

## Methods

### Respondents

Respondents met the following inclusion criteria: (1) age 18–25 years; (2) US resident; (3) self-reported diagnosis by a physician or other medical provider of one or more DSM-5^24^ mental health conditions; and (4) self-reported disability attributed specifically to their mental health disorder.^5^ Of 1112 persons who expressed interest in the study, 141 did not meet inclusion criteria, and 4 did not consent to participate, leaving a total of 967 respondents. Power calculations showed that the minimum sample size required to detect medium-sized effects (*f*2 = 0.15) for a 9 variable model would require 90 respondents, indicating adequate statistical power.^25^

### Procedures

Respondents completed an anonymous, online survey programmed in Qualtrics, with a $10 incentive, from March 26 to June 4, 2021. They were recruited from across the USA via social media, email, and web announcements posted by youth-focused behavioral health advocacy organizations such as Youth MOVE National, the National Federation of Families, the University of Massachusetts Transitions to Adulthood Center for Research, the UCLA Center for Mental Health in Schools and Student/Learning Supports, and the University of Illinois Chicago Center on Mental Health Services Research and Policy. The study was approved by the University of Illinois Chicago Institutional Review Board.

### Measures

Current mental health status was assessed using 3 well-known, validated screening instruments used successfully with young adults. Screening for MDD used the Patient Health Questionnaire-9 (PHQ-9),^26^ comprised of nine, 4-point Likert-scaled items measuring the frequency of depressed mood over the past 2 weeks, with a Cronbach’s alpha of 0.83^27^ and a cutoff of ≥ 10.^28^ GAD was assessed using the generalized anxiety disorder questionnaire-7 (GAD-7),^29^ containing seven, 4-point Likert scaled items measuring the frequency of anxiety symptoms over the past 2 weeks, with a Cronbach’s Alpha of 0.89^[30]^ and a cutoff of ≥ 10.^31^ PTSD was assessed with the Abbreviated PTSD Checklist-Civilian Version (PCL-C),^32^ including six, 5-point Likert scaled items measuring key PTSD symptoms related to any type of trauma over the past month, with a Cronbach’s Alpha of 0.91^33^ and a cutoff of ≥ 14.^34^ Physical health status was assessed using the single-item self-rated health (SRH) measure.^35^

Disruptions to normative life transitions were assessed with a measure created for this study called the Young Adult Disrupted Transitions Assessment^36^ (see copy in online supplement). Based on previous studies of transition to adulthood,^37–39^ thirteen “yes/no” statements described interruptions related to education completion (“I stopped attending high school, college, vocational classes”), launching an employment career (“I wasn’t able to get a job when I wanted to”), attaining residential independence (“I wasn’t able to move into my own place when I wanted to”), and establishment of marriage or other intimate partner relationship (“My marriage or intimate relationship ended”). Respondents were asked to select all “life interruptions you have experienced since the pandemic began in March 2020.” Dependent variables were 4 dichotomous (0/1) measures representing transition interruptions in each life area, coded as “1” if respondents endorsed one or more statements in that life area and as “0” otherwise.

Also collected was demographic information (age, race, ethnicity, gender, residential status), and social determinants (household income, social connections, community participation). Self-reported psychiatric diagnoses were coded in the following hierarchy: schizophrenia spectrum disorder, bipolar disorder without schizophrenia, depressive disorder without schizophrenia or bipolar disorder, anxiety disorder without schizophrenia, bipolar disorder, or depressive disorder, and all other disorders. Impairment due to mental health disorder was defined, following Davis and Koroloff,^5^ as ever enrolled in special education; used college disability services; received residential or psychiatric inpatient treatment; received public mental health services; encountered difficulty functioning in school, work or family life; and/or received public disability benefits. Pandemic barriers and social determinants were assessed with the Epidemic-Pandemic Impacts Inventory (EPII)^40^ consisting of dichotomous yes/no responses to 92 pandemic-related experiences across multiple life domains including economic security, work life, home life, social activities, and community connectedness.

### Statistical analysis

Descriptive statistics were computed for demographic information, the four types of transition disruptions, and the three mental health screening instruments. Multivariable logistic regression models for each transition disruption included GAD, MDD, and PTSD separately. All models controlled for race, Latinx ethnicity, gender, household income, and self-rated physical health. Each model also included 3 additional variables identified in prior studies of emerging adults in the general population to be associated with transition disruptions during the pandemic. For transition to residential independence models the 3 variables were residing with parents, living with other relatives, and change in number of co-residents.^20,21^ For formation of intimate partner relationships models the 3 factors were feeling lonely or isolated, having a current romantic relationship, and being prevented from developing new relationships due to pandemic restrictions.^23^ For establishment of careers through employment models, the 3 variables were difficulty with the transition to remote working, sleep problems, and increased consumption of junk food or overeating.^19,22^ For educational disruption models, the 3 factors were poor experiences with remote instruction, lack of technology for online classes, and inability to participate in sports or organized social groups.^19^

## Results

Table 1 presents characteristics of the 967 study respondents. Most identified as White (63.2%) with 36.8% Non-White. Half identified as female (49.0%, 474/967), one-third (34.6%, 335/967) identified as Latinx/Hispanic, and 34.9% resided with parents. Their average age was 22.1 ± 2.3 years. Mean self-reported health was 3.1 ± 0.8 indicating “fair” physical health.

**Table 1:** Characteristics of young adults (age 18–25) with pre-pandemic serious mental health conditions (*N* = 967)

	Total *N* = 967	Total %
Gender		
Male Female Non-binary, non-conforming, fluid, queer	476 474 17	49.2% 49.0% 1.8%
Race		
White Black Asian American Indian/Alaskan Native Native Hawaiian/Pacific Islander Other nonwhite	611 199 78 64 14 1	63.2% 20.6% 8.1% 6.6% 1.4% 0.1%
Latinx	335	34.6%
Annual household income		
$0–19,999 $20,000–49,999 $50,000–99,999 $70,000 +	42 373 300 252	4.3% 38.6% 31.0% 26.1%
Residing with parent(s)	337	34.9%
Residing with other relatives	72	7.4%
Change during pandemic in number of co-residents	321	33.2%
Pandemic restrictions on new friendships	608	62.9%
Feeling lonely or isolated during pandemic	135	14.0%
Currently in a romantic relationship	409	42.3%
Too much junk food or overeating during pandemic	433	44.8%
Sleep problems during pandemic	642	66.4%
Difficulty transitioning to working remotely during pandemic	437	45.2%
Unable to engage in sports or organized social groups during pandemic	671	69.4%
Poor experiences with remote classroom instruction during pandemic	413	42.7%
Lack of technology for online learning during pandemic	392	40.5%
Screened positive for major depressive disorder (PHQ > = 10)	789	81.6%
Screened positive for generalized anxiety disorder (GAD > = 10)	566	58.5%
Screened positive for post-traumatic stress disorder (PCL-C > = 14)	826	85.4%
	Mean ± SD	Median
Self-rated physical health	3.1 ± 0.8	3.0
Age, years	22.1 ± 2.3	22.0

*SD*, standard deviation, *PHQ*, Patient Health Questionnaire 9 item scale (Kroenke et al., 1999); *GAD*, generalized anxiety disorder 7 item scale (Spitzer et al., 2006); *PCL-C*, post-traumatic checklist – civilian 6 item scale (Lang & Stein, 2005)

Regarding pre-pandemic DSM diagnosis, 8.0% (*n* = 77/967) reported being diagnosed with schizophrenia or schizoaffective disorders; 5.6% (54/967) reported bipolar disorder; 19.3% (187/967) reported depressive disorder; and 67.1% (649/967) reported anxiety disorder. Regarding impairment *specific to their mental health condition*, 31.9%, reported being unable to work or attend school regularly due to a mental health condition, 23.9% had been psychiatrically hospitalized, 31.9% had been unable to work or attend school on a regular basis; 22.2% received residential treatment, 15.7% received public mental health services, 20.9% received accommodations from a college or university Disability Services office, 10.1% (98/967) reported receiving public disability benefits, and 6.6% had been enrolled in special education services.

**Figure 1:**
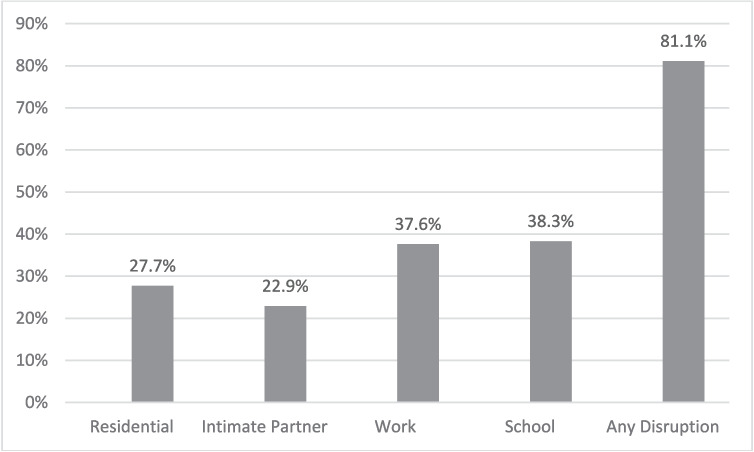
Transition disruption prevalence by type among young adults (18–25 years) with pre-pandemic mental health conditions (*N* = 967)

Figure 1 presents the prevalence of transition disruptions. The large majority (81.1%, 784/967) reported one or more disruptions, including completing formal education (38.3%, 370/967), launching an employment career (37.6%, 364/967), establishing residential independence (27.7%, 268/967) and forming an intimate partner relationship (22.9%, 221/967). In addition, large proportions screened positive for MDD (81.6%, 789/967), for PTSD (85.4%, 826/967), and for GAD (58.5%, 566/967).

Tables 2, 3, and 4 present models predicting the occurrence of specific types of transition disruptions and their association with the three types of psychiatric disorders separately. Models included demographics and social determinants, as well as factors found to be associated with specific transition disruptions during the pandemic. As shown in Table 2, MDD was associated with disruption in establishing intimate partner relationships; those meeting criteria for MDD were over one-and-one-half times as likely to report this disruption. Respondents who identified as Black or “other” non-White race were less likely to experience intimate partner disruption than those who identified as White. Those with higher household incomes and those feeling lonely or isolated were more likely to experience this disruption than their counterparts.

**Table 2:** Disrupted transitions associated with major depressive disorder and other factors for emerging adults age 18–25 (*N* = 967)

Respondent characteristic	Type of transition disruption^1^			
	Residential independence	Intimate partner relationship	Establishment of employment career	Completion of formal education
	Odds ratio [95% CI]	Odds ratio [95% CI]	Odds ratio [95% CI]	Odds ratio [95% CI]
Screened positive for major depressive disorder^2^	1.01 [0.70, 1.47]	1.66 [1.07, 2.57]*	1.04 [0.73, 1.48]	1.20 [0.85, 1.71]
Gender				
Male Female Non-binary, non-conforming, fluid, queer	Ref 1.31 [0.98, 1.75] 1.17 [0.40, 3.43]	Ref 0.90 [0.66, 1.23] 0.70 [0.19, 2.59]	Ref 1.02 [0.77, 1.34] 2.98 [1.06, 8.40]*	Ref 1.22 [0.93, 1.60] 1.59 [0.59, 4.30]
Race				
White Black Other non-white	Ref 0.58 [0.38, 0.87]** 1.05 [0.71, 1.55]	Ref 0.66 [0.43, 0.99]* 0.50 [0.31, 0.82]**	Ref 0.79 [0.56, 1.12] 0.88 [0.60, 1.29]	Ref 1.01 [0.71, 1.43] 1.20 [0.83, 1.73]
Latinx	1.17 [0.85, 1.60]	1.16 [0.83, 1.63]	1.54 [1.15, 2.05]**	1.24 [0.92, 1.65]
Self-rated health	1.08 [0.92, 1.27]	0.97 [0.82, 1.16]	1.12 [0.95, 1.31]	0.73 [0.63, 0.86]***
Household income	1.00 [0.93, 1.07]	1.09 [1.01, 1.17]*	0.92 [0.86, 0.98]*	1.05 [0.98, 1.12]
Residing with parent(s)	1.08 [0.79, 1.46]			
Residing with other relatives	1.32 [0.78, 2.26]			
Change in number of co-residents	1.06 [0.78, 1.45]			
Pandemic restrictions on new friendships		0.80 [0.56, 1.10]		
Feeling lonely or isolated		1.67 [1.10, 2.54]*		
Currently in a romantic relationship		0.76 [0.55, 1.04]		
Too much junk food or overeating			1.06 [0.80, 1.40]	
Sleep problems			1.57 [1.16, 2.11]**	
Difficulty with transition to remote work			1.59 [1.21, 2.08]***	
Poor experiences with remote learning				1.26 [0.96, 1.66]
Lacked technology for online learning				1.23 [0.94, 1.61]
Unable to do engage in sports or organized social grps				0.74 [0.56, 0.99]*

^1^Transition coded 1 if disrupted, 0 not disrupted

^2^Patient Health Questionnaire-9 (Kroenke et al., 1999)

*CI*, confidence interval; *p < 0.05, **p < 0.01, ***p < 0.001

**Table 3:** Disrupted transitions associated with generalized anxiety disorder and other factors for emerging adults age 18–25 (*N* = 967)

Respondent characteristic	Type of transition disruption^1^			
	Residential independence	Intimate partner relationship	Establishment of employment career	Completion of formal education
	Odds ratio [95% CI]	Odds ratio [95% CI]	Odds ratio [95% CI]	Odds ratio [95% CI]
Screened positive for generalized anxiety disorder^2^	1.73 [1.27, 2.36]***	1.42 [1.02, 1.96]*	1.83 [1.37, 2.43]***	1.12 [0.85, 1.48]
Gender				
Male Female Non-binary, non-conforming, fluid, queer	Ref 1.31 [0.98, 1.76] 1.12 [0.38, 3.32]	Ref 0.91 [0.66, 1.24] 0.69 [0.19, 2.55]	Ref 1.02 [0.77, 1.34] 2.85 [1.01, 8.04]*	Ref 1.22 [0.93, 1.60] 1.57 [0.58, 4.27]
Race				
White Black Other non-white	Ref 0.63 [0.42, 0.95]* 1.15 [0.78, 1.71]	Ref 0.66 [0.44, 1.01] 0.52 [0.32, 0.86]*	Ref 0.86 [0.61, 1.23] 0.95 [0.65, 1.41]	Ref 1.01 [0.71, 1.43] 1.21 [0.84, 1.76]
Latinx	1.10 [0.80, 1.51]	1.14 [0.81, 1.59]	1.46 [1.09, 1.95]*	1.23 [0.91, 1.64]
Self-rated health	1.13 [0.96, 1.33]	0.99 [0.83, 1.18]	1.17 [0.99, 1.38]	0.74 [0.63, 0.86]***
Household Income	0.99 [0.92, 1.06]	1.08 [1.00, 1.16]*	0.91 [0.85, 0.97]**	1.05 [0.98, 1.11]
Residing with parent(s)	1.17 [0.86, 1.59]			
Residing with other relatives	1.39 [0.82, 2.38]			
Change in number of co-residents	1.04 [0.77, 1.42]			
Pandemic restrictions on new friendships		0.83 [0.60, 1.14]		
Feeling lonely or isolated		1.65 [1.09, 2.50]*		
Currently in a romantic relationship		0.78 [0.57, 1.08]		
Too much junk food or overeating			1.05 [0.79, 1.39]	
Sleep problems			1.51 [1.12, 2.04]**	
Difficulty with transition to remote work			1.53 [1.16, 2.01]**	
Poor experiences with remote learning				1.27 [0.96, 1.67]
Lacked technology for online learning				1.23 [0.94, 1.60]
Unable to do engage in sports or organized social grps				0.74 [0.56, 0.99]*

^1^Transition coded 1 if disrupted, 0 not disrupted

^2^Generalized anxiety disorder questionnaire-7 (Spitzer et al., 2006)

*CI*, confidence interval; *p < 0.05, **p < 0.01, ***p < 0.001

**Table 4:** Disrupted transitions associated with post-traumatic stress disorder and other factors for emerging adults age 18–25 (*N* = 967)

Respondent characteristic	Type of transition disruption^1^			
	Residential independence	Intimate partner relationship	Establishment of employment career	Completion of formal education
	Odds ratio [95% CI]	Odds ratio [95% CI]	Odds ratio [95% CI]	Odds ratio [95% CI]
Screened positive for post-traumatic stress disorder^2^	0.82 [0.55, 1.24]	1.86 [1.09, 3.17]*	1.05 [0.71, 1.56]	1.54 [1.03, 2.31]*
Gender				
Male Female Non-binary, non-conforming, fluid, queer	Ref 1.31 [0.98, 1.75] 1.19 [0.41, 3.48]	Ref 0.90 [0.66, 1.23] 0.68 [0.18, 2.50]	Ref 1.02 [0.77, 1.34] 2.97 [1.05, 8.39]*	Ref 1.22 [0.93, 1.60] 1.55 [0.57, 4.19]
Race				
White Black Other non-white	Ref 0.56 [0.37, 0.85]** 1.03 [0.70, 1.52]	Ref 0.67 [0.44, 1.01] 0.52 [0.32, 0.86]*	Ref 0.79 [0.56, 1.13] 0.88 [0.60, 1.29]	Ref 1.03 [0.73, 1.47] 1.25 [0.86, 1.81]
Latinx	1.18 [0.86, 1.62]	1.16 [0.83, 1.62]	1.53 [1.15, 2.05]**	1.22 [0.91, 1.63]
Self-rated health	1.08 [0.92, 1.27]	0.97 [0.82, 1.16]	1.12 [0.95, 1.31]	0.74 [0.63, 0.86]***
Household Income	1.00 [0.94, 1.08]	1.07 [1.00, 1.16]	0.92 [0.86, 0.98]*	1.04 [0.97, 1.11]
Residing with parent(s)	1.08 [0.80, 1.46]			
Residing with other relatives	1.32 [0.77, 2.24]			
Change in number of co-residents	1.07 [0.79, 1.46]			
Pandemic restrictions on new friendships		0.81 [0.59, 1.12]		
Feeling lonely or isolated		1.66 [1.10, 2.53]*		
Currently in a romantic relationship		0.79 [0.57, 1.09]		
Too much junk food or overeating			1.06 [0.80, 1.40]	
Sleep problems			1.57 [1.16, 2.11]**	
Difficulty with transition to remote work			1.59 [1.22, 2.09]***	
Poor experiences with remote learning				1.27 [0.96, 1.67]
Lacked technology for online learning				1.23 [0.94, 1.61]
Unable to do engage in sports or organized social grps				0.74 [0.55, 0.99]*

^1^Transition coded 1 if disrupted, 0 not disrupted

^2^Abbreviated PTSD Checklist-Civilian Version (Lang & Stein, 2005)

*CI*, confidence interval; *p < 0.05, **p < 0.01, ***p < 0.001

Table 3 shows that those screening positive for GAD were more likely to experience disruption in achieving residential independence, establishing intimate partner relationships, and launching careers through employment. In addition to GAD, disruption in establishing residential independence was less likely among those who identified as Black compared to White, with no other model variables significant. In addition to GAD, disruption in establishing intimate partner relationships was more likely among those feeling lonely or isolated, and those with higher household incomes, but less likely among those who identified as “other” non-White race, compared to those who identified as White. In addition to GAD, disruption in the transition to careers through employment was more likely among those who reported being Latinx, identified their gender as non-binary/non-conforming/fluid/queer, experienced sleep problems, and had difficulty transitioning to remote working, and less likely among those reporting higher household income compared to those reporting lower income.

Table 4 shows that screening positive for PTSD was associated with intimate partner disruption and educational disruption. In addition to PTSD, disruption in the transition to intimate partner relationships was also more likely among those who reported feeling lonely or isolated and was less likely among those who identified as “other” non-White race compared to those who identified as White. In addition to screening positive for PTSD, disruption in education completion was less likely among those reporting better physical health and those unable to engage in organized sports and social groups.

## Discussion

This is the first study to show that poor current mental health is associated with specific types of transition disruptions for US young adults with pre-pandemic disabling mental health disorders. Disruption in one or more transitions was reported by over 80%, with more than a third reporting delays in launching careers or in completing formal education, and around a quarter reporting disruptions in establishing residential independence or in forming marital or other intimate partner relationships. Models that were tested explicitly incorporated social determinants of health to expand the field’s understanding of young adult mental health during the pandemic. High rates of transition disruption were associated with social determinants previously shown to influence young adults’ physical and mental well-being in the general population, including income, social connections, and community participation.^41,42^

Study respondents also reported exceptionally poor current mental health, with over 80% screening positive for MDD or PTSD, and over half for GAD. These high levels of mental health distress exceed those reported in population surveys of US high school^43,44^ and college students.^45,46^ Moreover, high rates of transition disruption and poor mental health support the narrative that COVID has been a collective trauma impacting US young adults.^47^ Additional research is needed to better understand the duration, course, and outcomes associated with such high rates of collective trauma for young adults with disabling mental health conditions.

Turning to specific associations, both depressive symptoms characteristic of MDD and feelings of loneliness or isolation were associated with disruptions in establishing intimate partner relationships. Social context is a well-recognized social determinant,^48^ and marginalized youth, such as those living with a disability, face multiple forms of social exclusion which heighten their risk of poor transition outcomes.^49^ A recent literature review found loneliness and social isolation to be associated with high depression severity among youth with pre-existing mental health conditions,^50^ pointing to the need to both reduce isolation and treat depressive symptoms. There is evidence that use of the internet and digital apps by young people has mitigated the impact of pandemic-related loneliness and poor mental health.^51,52^ This knowledge can be applied to newly developed interventions designed for young adults with pre-existing mental health conditions.^53^

High levels of anxiety indicating possible GAD, and symptoms characteristic of diagnosed PTSD, were associated with multiple types of pandemic-related disruptions, including failure to achieve residential independence, launch careers through employment, complete formal schooling, or establish intimate partner relationships. The social determinant of community context was evident in the association between education disruption and inability to participate in sports and organized social groups.^54^ While high prevalence of GAD and PTSD underscores the need for intervention and treatment, high rates of treatment discontinuation and low treatment access have been documented as young people move from child to adult mental health service systems.^55^ As a social determinant, barriers to accessing mental health care were exacerbated by pandemic lock-down orders, travel bans, and the switch to virtual behavioral health service delivery.^56,57^ This points to the need for evidence-based interventions that promote treatment access and engagement in this group of young people.^58^ Study findings regarding social determinants’ impact on young adult transitions also support the need for interdisciplinary collaboration between the fields of social work, education, psychiatry, psychology, family therapy, and rehabilitation counseling.^59–61^

Interestingly, study respondents who identified as Black or as members of other diverse cultures were less likely to experience disruption in transitions to residential independence and to establishment of intimate partner relationships. This may indicate the protective factor of culture in maintaining young people’s residential independence and romantic relationships. In one study, young adults who identified as racial minorities were less likely than their white counterparts to return to the family home during the pandemic.^62^ In another study, those in intimate relationships who identified as Black reported less decline in their functioning as couples compared to their white counterparts.^63^ Youth from Latinx, Black, Asian, and other diverse communities may have more established coping strategies that enable them to be psychologically resilient in the face of COVID-19. For example, Black youth’s connections to family, religious communities, and school support have been shown to buffer pandemic stressors and promote their mental health and well-being.^64,65^ Similarly, Asian American youth’s bi-cultural identity integration and ethnic-racial socialization were found to attenuate effects of pandemic-related racial discrimination on their mental health.^66,67^ In a related vein, Munson and colleagues^68^ found that stronger ethnic identity among Black, Latino/a, and multiracial young people living with serious mental illness was linked to greater feelings of personal recovery. Ways that young adults from diverse communities use adaptive coping, bi-cultural identity integration, ethnic-racial socialization, and community support to buffer pandemic stressors and maintain psychological resilience during global health emergencies deserve further exploration.

Study findings also confirm those of prior research on the impact of the pandemic on transitions of young adults in the general population. This study found that feeling lonely or isolated was associated with failure to forge intimate partner relationships, as did Dotson and colleagues.^23^ Study findings that young people’s sleep disruptions and problems adjusting to remote working conditions were associated with employment difficulties echoes results of the research of Hoyle and Davisson.^22^ Finally, the association between career disruption and young people’s identification as gender non-binary/non-conforming/fluid/queer confirms findings of Alessi and colleagues.^70^ These results suggest that the pandemic challenges confronted by this study’s respondents were similar to those of their counterparts in the general population.

### Limitations

One study limitation is that data come from respondents with access to hardware and technology needed to complete the online survey, and may not apply to emerging adults who lack these resources. Second, study data rely on self-report, which is subject to biases such as recall, forgetting, and positive response. Third, screening measures were used rather than diagnoses by clinicians, which may have over- or under-estimated prevalence of GAD, MDD, and PTSD. Fourth, it is impossible to say whether the transition disruptions reported were caused by the pandemic, pre-existing mental health conditions, social determinants, or other factors. Finally, the analysis did not include substance use and criminal justice involvement which could have influenced the associations that were examined.

## Implications for Behavioral Health

Study findings regarding associations between poor mental health and transition disruptions, and the influence of certain social determinants, can be used in the field of behavioral health. The importance of social determinants underscores the value of interdisciplinary, integrated care models that address young people’s needs related to economic security, education, social connection, secure housing, and community participation, along with mental health treatment.^55,71^ New models can build on culturally diverse young adults’ strengths stemming from adaptive coping, bi-cultural identity integration, and ethnic-racial socialization to help them buffer stressors and promote well-being.^65,67^ Moreover, developmentally appropriate peer support^72,73^ and use of internet, digital apps, and social media^73,74^ can increase access to emotional support and address loneliness and isolation that negatively impact mental health and disrupt life transitions. Training in mental health self-management skills^75,76^ can be integrated into treatment, along with strategies to help young people navigate adult systems of care, education, and rehabilitation services.^77,78^ Finally, the transition disruption assessment used in this study may be helpful for mental health professionals seeking to better understand young adults’ transition experiences. Young people may be more willing to discuss changes in their lives rather than their mental health, especially when beginning to receive services.

Since poor mental health may have aggravated already-challenging transitions for this group of young people, comparative studies of young people with and without pre-pandemic mental health disorders can help to understand the additional challenges faced by the former group and how to address them. Also needed is research on the long-term effects of pandemic-related disruptions as well as implementation and evaluation of supports for transition age individuals.^79^ In particular, longitudinal research showing change over time in mental health distress, achievement of expected life course transitions, positive and negative impacts of social determinants, and use of services and supports is needed to guide the development of programmatic and system responses to support these young people.

## Supplementary Information

Below is the link to the electronic supplementary material.

Supplementary file1 (DOCX 14 KB)
