# Supplementary material for: Social Determinants, Mental Well-Being, and Disrupted Life Transitions Among Young Adults with Disabling Mental Health Conditions
Source: J Behav Health Serv Res. 2025 Jan 13;52(2):263–81. doi: 10.1007/s11414-024-09924-0 (PMC11996939; doi:10.1007/s11414-024-09924-0)
Supplement: Supplementary file 1 — Supplementary file1 (DOCX 14 KB) [file 11414_2024_9924_MOESM1_ESM.docx]

**Social Determinants, Mental Wellbeing, and Disrupted Life Transitions Among Young Adults with Disabling Mental Health Conditions**

**Online Supplement**

**Young Adult Disrupted Transitions Assessment**

Instructions: The COVID-19 pandemic has disrupted many people's lives in a number of ways. Please choose which of the following life interruptions you have experienced since the pandemic began in March 2020. Please select all that apply.

1. I stopped attending my high school/college classes.

2. I stopped attending vocational classes.

3. I wasn’t able to get a job when I wanted to.

4. I was let go or laid off or quit my job.

5. I couldn’t find a romantic partner/someone to be intimate with/boyfriend or girlfriend.

6. I wasn’t able to move forward with my plans to get married.

7. My marriage or intimate relationship ended.

8. I wasn’t able to move into my own place when I wanted to.

9. I had to move out of my own place before I wanted to.

10. I had to live with parents or other family members when I didn’t want to.

11. I left high school/college without graduating.

12. I didn’t start college or vocational training when I had planned to.

13. None of these apply to me

Scoring: Education completion disruption = 1, 2, 11, or 12; Residential independence disruption = 8, 9, or 10; Employment disruption = 3 or 4; Intimate partner relationship disruption = 5, 6, or 7

Citation: redacted
